# Perimesencephalic subarachnoid haemorrhage: A case study

**DOI:** 10.4102/jcmsa.v3i1.245

**Published:** 2025-09-11

**Authors:** Esaias C.C. Koller, Joubert C. Steynberg, Zainub Jooma

**Affiliations:** 1 Department of Anaesthesia, Charlotte Maxeke Johannesburg Academic Hostpital, Johannesburg, South Africa; 2 Intensive Care Unit, Wits Donald Gordon Medical Centre, Johannesburg, South Africa; 3 Department of Anaesthesia, School of Clinical Medicine, Faculty of Health Sciences, University of the Witwatersrand, Johannesburg, South Africa; 4 Chris Hani Baragwanath Academic Hospital, Johannesburg, South Africa

**Keywords:** perimesencephalic, subarachnoid, haemorrhage, non-aneurysmal, case study

## Abstract

**Introduction:**

Spontaneous subarachnoid haemorrhage (SAH) is caused by a ruptured aneurysm in most cases. In 15% of cases, no vascular abnormality can be found. This subset is classified according to blood distribution, which can be perimesencephalic or non-perimesencephalic. Perimesencephalic subarachnoid haemorrhage (PM-SAH) is the least common phenomenon. It has a favourable prognosis with negligible long-term complications. A high index of suspicion is needed to identify this group of patients. This report highlighted the current consensus for managing these patients. It also highlighted the need for further research as no cause has been identified.

**Patient presentation:**

A 35-year-old male developed PM-SAH during physical exertion. He had no risk factors and was well below the described age of being in the 6th decade of life.

**Management and outcome:**

The patient was admitted to an intensive care unit and had digital subtraction angiography performed within the prescribed 24 h–48 h. This modality digitally subtracts radiopaque structures such as bones to enhance the visibility of blood vessels. Treatment was symptomatic, and a course of nimodipine was completed. The clinical course was uncomplicated, and the patient recovered fully.

**Conclusion:**

Perimesencephalic subarachnoid haemorrhage is a benign condition once aneurysmal causes have been excluded. As PM-SAH follows a diagnosis of exclusion, the initial monitoring and management follow the same principles of an aneurysmal SAH.

**Contribution:**

In this uncommon phenomenon of PM-SAH described in this case, an even more uncommon anatomical variant, namely a superior cerebellar artery fenestration, has been identified. This possibly highlights the role that vascular anatomical variants play in this condition, where no cause has been identified.

## Introduction

Subarachnoid haemorrhage (SAH) is a devastating condition accounting for 5% of strokes and occurs at a relatively young age.^1,2^ Aneurysmal subarachnoid haemorrhage (aSAH) is a spontaneous event but in a small subset, no vascular abnormality can be found. The distribution of blood can be around the midbrain that is perimesencephalic (PM-SAH) or exceeding beyond the former known as non-perimesencephalic (NPM-SAH). Angiography or autopsy is needed to diagnose the presence of a ruptured or unruptured aneurysm. An estimate of 1 in 50 people have an unruptured intracranial aneurysm, but the incidence of SAH in this population is still unclear.^2,3^

## Patient presentation

A 35-year-old gentleman went on a regular morning social mountain bike ride. He is a non-smoker and only takes simvastatin 20 mg orally daily for hypercholesterolaemia. During a strenuous uphill climb, he suddenly experienced an immense pressure in his neck that radiated into the occiput. This was followed by an intense headache. He experienced a slight loss of balance, so he went to the closest place of safety and got off his bike. At this point, he experienced paraesthesia in his hands, nausea and neck stiffness. He called for help and was admitted to a medical centre. On arrival, he received paracetamol 1 g IV and granisetron 1mg IV. This immediately alleviated the symptoms. He suffered from muscular spasms in his upper back, and he regarded his stiff neck to be from the spasms. Magnetic resonance imaging (MRI) of the cervical spine was ordered together with an MRI of the brain because of the headache. The attending physician was alerted that the patient had an SAH located in the basal cisterns around the brainstem. The patient went back to radiology to perform a computed tomography angiogram (CTA) of the cerebral vessels. No aneurysm was noted. The patient was admitted to the intensive care unit (ICU) and started on the SAH protocol. This included 4-hourly 60 mg oral nimodipine, adequate analgesia, anti-emetics and a stool softener as needed. Theatre was booked to perform an urgent digital subtraction angiography (DSA). Invasive monitoring was placed in theatre, and the goal was to perform a haemodynamically stable general anaesthetic while maintaining cerebral perfusion by blood pressure control and blood flow. A modified rapid sequence induction with sufentanil, propofol and rocuronium was performed, and the airway was secured with an endotracheal tube. The neuroprotective goals were to avoid hypoxia and hypercapnia and maintain a normal serum glucose. Adequate analgesia was given to avoid sympathetic stimulation. The perioperative phase was maintained with desflurane in an oxygen and air mixture.

**FIGURE 1:**
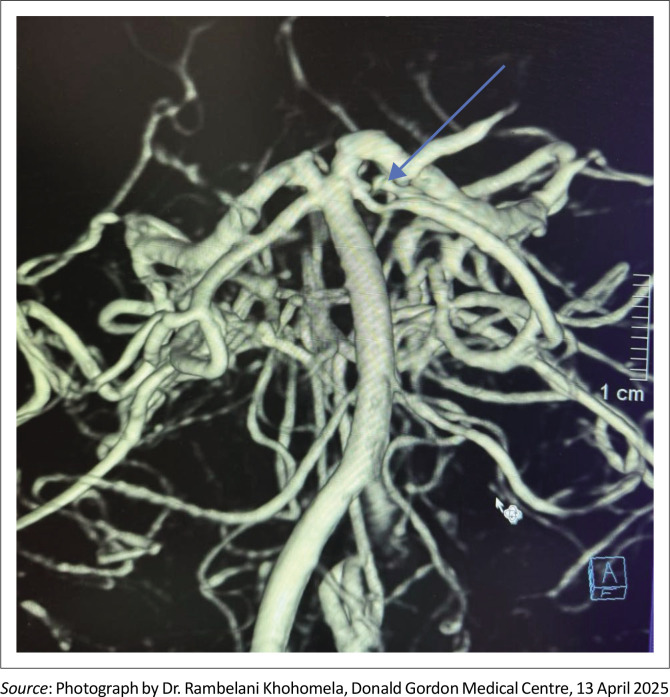
Left superior cerebellar artery fenestration (blue arrow).

The patient was found to have a left superior cerebellar artery fenestration on imaging with no cerebral aneurysm noted in the Circle of Willis. The imaging that was performed in theatre is provided in Figure 1. A superior cerebellar artery fenestration comprises a vascular segment that separates into two channels and then reunite distally to form a single lumen. This is a rare occurrence that stems either from partial obliteration of the primitive vessels during embryological development or incomplete union of the primitive arteries in the embryo. There have only been seven reported cases in the relevant English-language literature up until March 2023. Two cases were diagnosed on MR angiography, one case by DSA and four cases by CT angiography.^4^ Uchino et al. concluded that congenital weakness of the wall and haemodynamic stress of the fenestrated segment can lead to the formation of an aneurysm at the proximal end.^5^ If present, the aneurysm should become evident with serial imaging.

The patient had an uneventful 3-day ICU stay with no systemic effects from the nimodipine, maintaining systolic blood pressure between 120 mmHg and 145 mmHg. In the ward on day 5, the patient experienced severe back pain accompanied with a headache. An urgent CT brain was performed to rule out re-bleeding. No bleeding or signs of raised intracranial pressure was noted. The blood in the basal cisterns previously seen had completely resolved. The pain was regarded as muscular spasms from the lumbar spine into the lower limbs. Physiotherapy was consulted. The patient was discharged on day 6. A repeat CTA was performed on day 12, which still showed no aneurysm. The patient completed 21 days of nimodipine and had an uneventful recovery.

## Discussion

Subarachnoid haemorrhage is a deep brain bleed that develops between the pia- and arachnoid-mater. It can originate either spontaneously or from a traumatic event. Spontaneous SAH has an incidence of 7–9 per 100 000.^6,7^ The majority is caused by an aneurysm (aSAH), but in roughly 15% of patients, no vascular abnormality can be found.^1,6,8,9,10,11^ The proportion that is non-aneurysmal (naSAH) can be further subdivided into perimesencephalic (PM-SAH), accounting for a third, and NPM-SAH, accounting for two-thirds.^1^ This relates the incidence of PM-SAH to 0.3–0.5 cases per 100 000 persons.^8^ The condition has been shown to be benign with minimal long-term complications.^6,8,9,10^

The mean age of onset is 53–56-years-old with a slight male predisposition.^6,12,13^ However, in China, women are more affected than men.^2^ Hypertension, smoking and diabetes have been shown to be risk factors.^8,12^ There has been no specific correlation between alcohol consumption and PM-SAH.^8^ Laukka et al. have shown that physical exertion was a triggering factor in 79% of patients.^12^ It is suggested that physical exertion causes venous hypertension, leading to an increased intrathoracic pressure, which blocks venous return through the internal jugular vein.^7,11^ With a PM-SAH, there are usually variations in the venous drainage system of the mesencephalon, especially a more primitive drainage system of the basal vein of Rosenthal.^6,7^

Presenting symptoms are usually transient and includes headaches, nausea and vomiting, neck pain and meningism, transient focal symptoms and rarely seizures.^7,11^ Focal signs include diplopia, cranial nerve VII fallout, medulla involvement and dysphagia. The respiratory nucleus can be involved, which alters breathing patterns.^8^

Derangement in admission serum biomarkers include hyperglycaemia and high serum glucose variability, because of the stress reaction of the condition. An elevated C-reactive protein and white cell count may predict a worse clinical outcome in naSAH.^9^ The presence of red cells in the cerebrospinal fluid (CSF) should raise suspicion in the patient presenting clinically with an SAH.^14^

The diagnosis for naSAH follows a diagnosis of exclusion. When a PM-SAH is suspected, CT or MRI is urgently required as the SAH will redistribute after 72 h and alter the bleeding pattern.^11^ The bleeding pattern for PM-SAH is defined as: haemorrhage in the perimesencephalic cisterns immediately anterior to the midbrain, possible extension of blood to the posterior interhemispheric fissure or base of the Sylvian ridge, possibly small amounts of sedimented blood in the ventricles (no frank blood) and absence of brain haematoma.^1,6,7,11,13^

If no aneurysm is detected on CTA, DSA must be performed within 24 h–48 h as it is vital to exclude aneurysmal causes.^8,13^ The procedure is carried out in angiography suite and vascular access is achieved via the femoral or brachial arteries. The vertebral artery, internal and external carotids are selectively catheterised and visualised with contrast. The projections are anterior-posterior, lateral, oblique and rotational as guided by the neurological intervention specialist.^13^ Radiopaque structures, that is bones and dense tissue, are removed via digital methods to accurately view the blood vessels.^8^

Digital subtraction angiography is currently the gold standard to exclude an aneurysm as it provides a more defined image in comparison to CTA by excluding surrounding tissues.^8,11^ Because of the invasiveness of the procedure, high-resolution CTA has gained popularity in recent years offering a high sensitivity and specificity. Recent evidence has favoured CTA as an acceptable alternative for DSA.^7,10,11^ Nonetheless, it is still widely accepted that DSA should be performed in the acute setting.^8^

Although the bleeding source remains unknown, it is suspected that naSAH is from venous origin with a subsequently lower bleeding velocity, which accounts for the lower distribution of the bleed.^6,9,12^ Another hypothesis is that it originates from the rupture of a perforating artery.^6,7,12,15^ However, none of these lesions have been reported in more than a few patients.^7^ The blood load for PM-SAH is lower than for aSAH or NPM-SAH.^9^ The pattern of a perimesencephalic haemorrhage does not exclude an aneurysm.^7^ Furthermore, the absence of an aneurysm on initial imaging also does not exclude an aneurysm as the vessel can contract post-bleeding and only become evident a few days later.

Patients presenting with SAH of unknown origin usually stay in the ICU for 2–4 days and require intensive neurological monitoring.^7,9,11^ The treatment is mainly symptomatic, which focuses on treatment for headache and nausea and monitoring of electrolyte imbalances, hydrocephalus, vasospasm and seizures.^7,11^ Most acute episodes of hydrocephalus resolves spontaneously and intervention is seldom required.^7,8,11^ Vasospasm is a recognised complication in SAH, which can lead to delayed cerebral ischaemia (DCI); however, symptomatic vasospasm in PM-SAH is rare.^6,7,11^ Vasospasm was previously treated by ‘Triple-H’ therapy including hypertension, hypervolemia and haemodilution. However, this therapy has been associated with increased cardiopulmonary complications because of excessive volume administration and the risk of ischaemia associated with anaemia.^11,16^ Currently, there are no randomised clinical trials to support this therapy.

Delayed cerebral ischaemia is not usually associated with PM-SAH. The reports that have described DCI were closely related to the DSA and were regarded as procedure-related ischaemia.^7^ Mensing et al. state that a convincing clinical picture of DCI in PM-SAH has not been reported, so there is no indication for using nimodipine.^7^ Haugh et al. also found that nimodipine had no difference in the rate of complications in patients where no aneurysm was detected on DSA.^6^ Anticoagulant drugs should also be avoided in the acute phase.^7^

Forced bed rest or restriction of activities is not necessary as the risk of complications is low.^6,7,8,11^ Patients have a normal life expectancy and no restrictions should be implemented on daily activity.^7,11^ A modified Rankin scale of 0–2 represents a favourable outcome for functional independence. Majority of patients with PM-SAH fall into this category.^6,11^ Mensing et al. state that patients can be discharged 24 h after ictus as the risk of symptomatic hydrocephalus then is negligible.^7^ However, most patients complete a 2–4 day in-hospital stay.^7^

In general, the clinical outcome for PM-SAH is favourable compared to aSAH. Long-term complications are minimal, and death has rarely been reported.^6,8,11,14^ Factors that are associated with a positive outcome include young age, good neurological condition at hospitalisation, no hydrocephalus and a perimesencephalic pattern rather than aneurysmal pattern.^15^ Re-bleeding is uncommon with a risk of 79 per billion over a lifespan of 80 years.^11^ There is a concern for cognitive decline as a long-term complication.^8,11^ Haemoglobin has local neurotoxic effects, and the overall iron (from haem) can cause secondary brain injury with poor cognitive outcomes.^6^ One study concluded that the risk for cognitive impairment as a long-term complication is negligible.^8^ There is a paucity of research on this topic and only includes small-sized study groups.^1,6,9,10,14,15^

The risk factors for aSAH and naSAH are comparable, but the clinical presentation and long-term outcome differ notably. No specific cause has been linked to PM-SAH, and the minimal long-term follow-up has been done. Further research is recommended with ample-sized study groups.
